# Reviewing the Evidence on Sibling Sexual Behaviour: Impact on Research, Policy and Practice

**DOI:** 10.1007/s11920-024-01487-3

**Published:** 2024-02-12

**Authors:** Kieran F. McCartan, Sophie King-Hill, Stuart Allardyce

**Affiliations:** 1 University of the West of England, Bristol, UK; 2 University of Birmingham, Birmingham, UK; 3 Lucy Faithfull Foundation, Bromsgrove, UK

**Keywords:** Sibling sexual abuse, Prevention, Treatment, Policy, Family, Professional practice

## Abstract

**Purpose of Review:**

This paper reviews recent research into sibling sexual behaviour (SSB). This is an emerging professional and community issue that binds together a limited evidence base across research, practice and policy in psychology, criminology, politics, social work and policy studies. The review will demonstrate that a multi-disciplinary, life course, family system approach is the most effective way of starting to develop interventions to prevent and respond to this issue.

**Recent Findings:**

SSB has previously been researched as a form of intrafamilial abuse or sibling incest. As a result of this SSB is poorly and inconsistently defined as a concept, meaning that research, practice and policy are sometimes at odds with each other and need to pull together to develop a cohesive framing of the issue. This means that a lot of older research needs to be contextualised in new emerging frames of thinking and ways of working. Current research emphasises the importance of understanding the role of the family system in creating conditions where SSB can occur and its central role in preventing and stopping it from occurring. The research also stresses the importance of professionals understanding the family context of SSB and has the confidence to identify and work proactively with families in a multi-agency and cross-disciplinary way.

**Summary:**

The prevention of, and response to, SSB requires a multi-level, multi-disciplinary approach. Successful prevention of and response to SSB are as much about the family system as it is about the attitudes, behaviours and experiences of the siblings impacted by the abuse.

## Introduction

Recently, there has been increased academic and policy interest in sibling sexual behaviour (SSB) [1••, 2••]. It is important to recognise that this does not mean that SSB is a new form of child sexual abuse and exploitation [3] and that this is a recognition of a long-standing issue rather than a new phenomenon. In discussing SSB, it has become apparent that it is an under-researched area, especially in comparison to other forms of sexual violence [1••, 2••]. Most intra-familial sexual abuse research focuses on father-daughter incest [4], and most peer-on-peer childhood sexual harm research is centred around non-familial relationships [5, 6•, 7]. Historically, up to the start of the twenty-first century, clinical and academic discussions focussed on ‘sibling incest’, a concept that typically minimises the harm caused by SSA [8]. This poses a challenge when developing evidence-based responses to this issue as the historical evidence base in relation to SSB is not as robust as that in other areas of sexual abuse (i.e., risk assessment, treatment, reintegration) [9, 10]. This means that policy and practice development is happening in parallel with an evolving evidence base. This article will draw together the existing, limited, evidence base related to SSB, highlighting what is unique about it compared to other forms of child sexual abuse.

Definitions of SSB focus on harmful sexual behaviour between children who self-identify as siblings have been known under the umbrella term of sibling sexual abuse [2••, 14]. Although this definition has merit, the term’harmful sexual behaviour’ includes behaviours that are inappropriate or problematic, which may be developmentally harmful for all involved but fall short of victimising intent or outcome [17•]. This may result in behaviours being labelled as abusive which would better be described as problematic: e.g. younger children who may be acting out with a sibling their own experience of victimisation or exposure to sexual experiences they are not developmentally ready for. Some definitions compound this by including a range of actions that may be abusive, but in some contexts, these may be better described as inappropriate or problematic (e.g., unwanted sexual references in conversation, indecent exposure, or forcing a child to view pornography) [18]. Some definitions emphasise age difference and/or use of force or coercion, but research to date suggests that power difference may be a better foundation for establishing whether sexual behaviour between siblings is abusive [19].

Studies of young people who have either displayed or experienced SSB use different definitions and are often describing different kinds of scenarios and contexts using the term ‘sibling sexual abuse’, some of which may fall short of typical definitions of child sexual abuse, and which would be better characterised as inappropriate or problematic sexual behaviour between siblings [2••]. This compromises the utility of research when informing decisions about live case situations. Given this, the term sibling sexual ‘behaviour’ rather than ‘abuse’ will be used in this paper to encompass the range of behaviours that are being referred to.

## Implications of the Complexity of Definitions

SSB is compounded by a number of challenges that have implications for detection and intervention. One of the key aspects of these challenges relate to the complexity of the multi-layered definitions of SSB in various fields. Although the scale and seriousness of this subject is increasingly recognised amongst researchers and practitioners, there is no universally agreed definition of SSB [1••, 2••]. This flows from two issues of complexity: lack of agreed definition of what constitutes a sibling relationship and a lack of professional consensus about what constitutes abusive sexual behaviour between siblings. However, it must be stated that this definitional nuance is not limited to SSB but to sexual abuse in general and violence against women and girls specifically, but unlike with SSB, these other examples have been debated and agreed in the UK with a majority consensus in place. That is to say that there is an operational definition, which is where sibling sexual behaviours, as a range of behaviours and harms, is lacking [1••, 2••, 12].

A further complexity surrounding SSB stems from the difficulty in defining what constitutes a sibling. There are many forms of sibling relationship, such as biological brothers and sisters, step-siblings, half-siblings, adoptive siblings, foster siblings, and social siblings—children not biologically or legally related but who have been brought up together or in close proximity and share an enduring bond. In some cultural and social contexts, extended family relationships exist that share many of the characteristics of what may be conceptualised as that between siblings [13]. Recognition of these complexities means that self-definition is now often foregrounded in defining what a sibling relationship is within a particular family and context [14].

How the behaviour itself is defined also required consideration. Some definitions of SSB focus on sexual contact between siblings that is experienced by a survivor as traumatic [15]. Although this will describe many scenarios involving SSB, for some children, the experience of trauma may not be obvious at the time and may only become apparent in later adolescence and adulthood [16]. Definitions focusing on trauma may therefore compromise safeguarding investigations where SSB is identified, as the child who has experienced harm may not conceptualise what has happened to them as harmful and/or may be asymptomatic [1••, 2••]. Some studies interviewing adult survivors identified sibling sexual behaviour that is significantly outside of the parameters of normative, developmentally appropriate sexual behaviour but does not seem to lead to trauma then or in later life [20•]. These ‘routine relationship types’ involve those in the sibling subsystem creating an intense shared world (sometimes within a context of parental neglect) [21], with the older siblings initiating sexual acts as part of daily life, while the younger ones tending to accept them and comply as they do in any other situation. Whether experienced as pleasurable, uncomfortable or painful, the sexual acts are seen as normative and acceptable by the children involved. Such situation challenge definitions around sibling sexual behaviour and the terms such as sibling incest and ‘sexual behaviour between siblings’ continue to be used by some researchers considering the unsettled nature of definitions in this area of child maltreatment [22]. Lack of agreed definitions means that studies looking at prevalence vary widely as they rely on differing definitions of what constitutes sibling sexual abuse. Population-based prevalence figures vary from 1.3% of children experiencing SSB [23] to 11% [24].

Another implication of lack of clear definition of SSB is that social workers and other professionals may underappreciate the abusive nature of certain sexual interactions between siblings, identifying them as normative or inappropriate, or conversely label age-appropriate sexual exploration between siblings and behaviour that is mutual but not harmful as abuse [25•]. SSB has often been underreported or dismissed by adults as ‘harmless sexual experimentation’ [11]. This is particularly the case where sibling sexual behaviour has been identified that does not meet traditional professional expectations of what constitutes child sexual abuse, even though they meet key criteria of definition of child sexual abuse (e.g. sexual contact between children where there are significant power differences between participants).

The complexity and nuance in defining SSB has fed into the development of a complicated and ill-defined evidence base. Literature searches in SSB highlight relatively recent articles and policies [1••]; however, the research evidence base is older than it initially appears. When considering the research evidence, a range of terms require exploration across a range of disciplines [1••, 2••] (see Table 1 for an example). However, it is useful to note that the terminology across disciplines is varied using terminology such as sibling incest as opposed to sibling sexual abuse (i.e., Anthropology [26], Social Work [27] and Sociology [28]). This indicates that to fully understand the nature and lived experience of SSB, a multi-disciplinary understanding is required [1••, 2••, 29] that takes account of the presence and impacts of vulnerabilities through an intersectional lens [30, 31].

**Table 1:** Alternate definitions of sibling sexual behaviour [1••]

• Sexual interaction between siblings	• Sibling sexual abuse	• Sibling sexual exploitation
• Sibling incest	• Problematic sexual behavior between siblings	• Sexual behaviours between siblings
• Sex between siblings	• Sexual curiosity between siblings	• Mutual sexual acts siblings
• Child sexual abuse among sibling	• Sexual activity between siblings	• Sexual exploration between siblings
• Brother-sister incest	• Sibling sexual experimentation	• Sexual dynamics siblings

The complicated picture adds to the understanding that SSB is a constellation of behaviours and that the abusive behaviour is just one of many sibling sexual interactions that can occur in the context of the family home [1••, 2••, 12]. This means that both the UK national and international evidence base is fluid and lends itself currently to informing guidance and limited policies and practices rather than a definitive framework [32–35]. Therefore, it is important to make sure that there is a shared definitions of SSB that will inform the research evidence base and improve practice and policy [12, 34]. If there are too many variations of definitions, whether by sector (i.e. in the UK; police, social work, youth services, the third sector) or by role (i.e. in the UK; prevention, treatment, risk assessment, risk management), this may lead to a disjointed system that is unable to effectively help young people and their families. Additionally, traditional sexual abuse theories and approaches (i.e. desistence, risk/needs/responsibility and strength-based approaches) have not been applied to SSB the way that they have with other special populations (i.e. females who offend, young people who sexually harm, individuals with a mental illness or intellectual inability who sexually harm] [9, 10]. The logic being that we are at the start of developing our evidence base around SSB and do not know the medium- and long-term implications on either sets of siblings (i.e. those that have harmed and those that have been harmed). This means that consideration has to be given to the realities of prevention, treatment, desistence, community integration and support for victims as well as those who have harmed and what is unique to SSB and what is not. Understanding of these issues will develop in respect to SSB and will come in time and so too will better linkage to other mainstream theories as well as the development of bespoke adaptions.

## The Influence of Family Dynamics

**Fig. 1:**
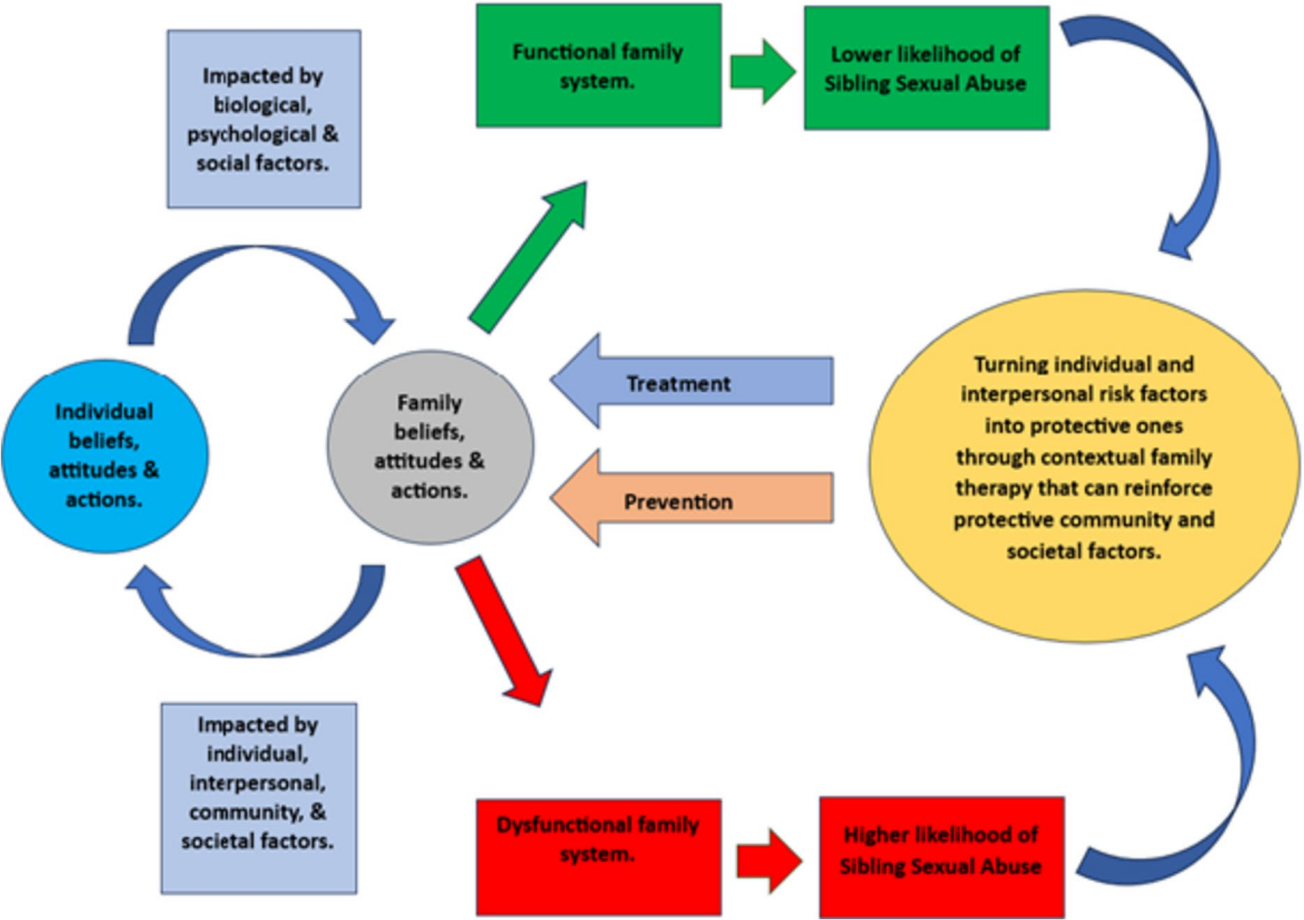
The interrelationship of family dynamics on the aetiology, prevention and response to sibling sexual abuse [1••, 36]

One of the unique factors in SSB is the role of the family in the abusive situation. The family system is a contextual factor that can either precipitate the abuse and/or help sustain the harmful behaviour when there is a wider culture of abuse and dysfunctionality. Similarly, healthy family dynamics can contribute to cessation and desistance of abuse [1••, 2••, 16, 36] (see Fig. 1).

The family system is an important domain in SSB, in addition to the child that has been harmed and the child that is being harmed; therefore, taking the family into account is essential in preventing and responding to SSB. This is highlighted via new mapping guidance for responding to SSB that has been developed in the UK [37••]. This indicates that shifting the conversation from the abusive behaviour to the contextual factors and focusing as much on the family environment are vital components. Accounting for the family dynamic, the individual’s psychology and behaviour and the relationship between the two siblings are all as important as each other in preventing and responding to SSB [38, 39••]. This indicates a different type of professional engagement that is longer, more intensive, more inclusive, more complex and potentially more costly, which means that SSB needs to be thought of as typically occurring within a culture of harmful sexual behaviour, rather than a abusive behaviour that triggers multi-agency responses focussing on individuals [1••, 36]. One example might be an adaption of contextual safeguarding for the family system and the services that surround them [6•, 7]. Traditional contextual safeguarding places the child at the centre of a range of personal, community and professionals’ systems all of which work together to reduce harm between children to ultimately help them integrate and fully function in the community [6•, 7]. From an SSB perspective, consideration should be given to the contextual safeguarding model and how the personal, familial (nuclear and extended family), peers, community and professional systems come together to support the family members affected by this issue.

It has been advocated in studies and literature that the focus of any successful intervention for SSA should be embedded within the family setting in a strength-based, restorative approach [1••, 2••, 51]. This approach requires listening to the voices of all of those involved from the outset to gain insight into the context in which they are situated. This insight is not only linked to the sexual behaviour itself but also to other contextual factors such as living arrangements, family dynamics and parenting, education, health and development and social context [37••]. This is advocated by the recently developed SSB mapping tool (SSBMT) developed in the UK, to aid practitioner thinking in relation to the complexities of this issue. It is firmly embedded within all these social structures, and gaining an adequate picture of these elements is crucial to designing successful interventions [2••]. This holistic gathering of information begins with the CYP and their families and is a key factor in long-term positive outcomes [2••, 49, 52]. This also links to the crucial nature of multi-agency working as all these differing aspects of family life need to be considered [2••, 49, 53, 54].

When considering this, it is important to also acknowledge the professional responses to disclosure of SSB. These reactions are linked to confidence, knowledge and skills and play an important role in what happens to CYP and their families. When lacking these key skills, professionals may minimise, catastrophise or exaggerate (to gain access to services) the behaviours of the CYP, all with potentially damaging effects to the CYP and their families [1••, 51] as these reactions, quite often, originate from a lack of knowledge and confidence specifically about understanding when sibling sexual behaviours are abusive [38, 52].

## The Impact of the Evidence Base on Professional Practice

Professional responses to SSB are a key contributor to successful outcomes for children who sexually harm their siblings, children who have been harmed and their family [1••]. Professionals involved with supporting families affected by SSB will need to make decisions that can have profound impact upon them (e.g., deciding whether children can continue to live together in the same household). This means that there is a responsibility placed on the professional to make sure that they are skilled and knowledgeable about the existing evidence, best practice and the potential impacts of their decisions. Successful professional engagement is also dependent upon the confidence of the professionals when addressing and planning the complex interventions that are required for SSB. However, in the UK, research has demonstrated that professional confidence is often low when considering harmful sexual behaviours of children and young people (CYP). This is further exacerbated in SSB due to the additional complexities that surround it. Despite this being a key aspect of positive outcomes, professional confidence varies greatly and is sporadic and inconsistent, and there is a vast amount of professional anxiety when presented with cases relating to SSB [1••]. Professional confidence in the UK also appears to derive from experience rather than specific training despite being a crucial element to the approach and outcomes when a family is experiencing SSB [2••]. This absence of confidence is also negatively combined with the lack of consistent national pathways, policies and structure which are required to plan robust interventions for families affected by SSB. UK-based research has demonstrated that professionals often feel underprepared when working in this sector [40]. This is one of the compounding factors that impact confidence around decision making, which is critical given the impact of some of the decisions that need to be made by professionals. These are then furthered by external issues such as a lack of knowledge and research in SSB and sporadic skills, experience and training [41•, 42]. Professionals are often internally influenced by their own self-efficacy and self-belief when making decisions in complex areas such as SSB [42]. Conversely, despite these factors that can compromise professional engagement with families affected by this issue, there is an inherent assumption in the UK that professionals dealing with SSB and its complexities are confident in this area, adding additional pressure to an already fraught and nuanced issue [41•, 43••]. The low confidence and dearth of training and research in SSB and how to conduct successful interventions lead to negative outcomes for CYP and their families who find themselves in this situation.

One of the ways that these issues of confidence can be addressed is via consistent training, increasing knowledge and therefore confidence in addressing SSB. A raise in confidence, via professional training, can increase well-planned professional interventions and ultimately leads to positive outcomes for those experiencing SSB and their families [43••, 44•, 45]. Planning and carrying out interventions in a consistent way is also heavily dependent upon effective multi-agency working. Again, due to the sporadic nature of interventions and the lack of a national, joined up, policy to address SSB, this is often piecemeal, leading to a ‘postcode lottery’ for those in this situation [1••]. The complexity that surrounds SSB requires a range of professional inputs. This is due to the differing elements of this work that is required for successful interventions to be planned. For example, work will need to be completed with the child that has harmed (that may also have been harmed themselves), the child that has been harmed and the wider family circle—often living in different homes. This is further complicated by a lack of shared language when working across agencies in this area [46–48]. This work requires a varied set of skills from professionals making effective multi-agency working a vital part of interventions [2••, 49, 50].

## Considerations for Policy

No streamlined national policies exist in relation to SSB in the UK context. This is due to the sporadic nature of research, practice and understanding in this field. Research indicates that some of the causal factors in relation to SSB relate to family dynamics and unresolved issues within families that impact on sibling relationships and development. The impact of SSB is also not exclusive to the child who has been harmed. Parents [55], non-abusing siblings [56] and even the sibling who has caused the harm [57] can be significantly impacted both at the time the abuse is identified and over their life course. Literature on resilience of survivors of child sexual abuse indicates that a range of family factors can improve outcomes for survivors, including emotional support from both parents, family stability, family connectedness and positive parenting [58]. All of these can be compromised in situations because of unresolved issues within the family. Siblings who have been harmed may also cut themselves off from potentially supportive family members as they grow older, because the family continues to be experienced as an emotionally unsafe place [20•, 59•]. Siblings may attempt to avoid contact with each other in adulthood because of unresolved issues, but events such as weddings and funerals can throw them together and become emotional minefields that cause stress for all members of the family [59•].

Typical responses in the UK to SSB when identified in childhood involve siloed services for family members: therapeutic work with the child who has experienced harm and assessment and interventions with child who has caused the harm to reduce risk. When such cases are identified in adulthood in the UK, individualised therapy may be available for the adult who was harmed in childhood. If the case is dealt with in the criminal justice system in the UK, ‘sex offender’ treatment will typically be offered [19]. What is typically lacking in this context, both in the child protection system and later when the children are adults, are interventions that support the whole family. Research suggests that responses to SSB are more effective when they engage with the family as a system rather than individualised, isolated responses to the child who has harmed and to the child who has been harmed [1••]. Family relationships as a whole need to be addressed and as far as possible repaired and restored whenever possible.

There have been some promising early indications that responses based upon some of the principles of restorative justice may be helpful for families in these situations [49]. Diversion from the courts to a family conferencing system in Australia [60] and a similar exemption committee scheme in Israel [61] all point in the direction not only of these approaches being robust, as far as is appropriate, in holding the child who has harmed to account, but also of their potential for more successful family restoration and rehabilitation. Such approaches stress the importance of understanding the family to be a ‘traumatised system’ and for any restoration work to be cognisant of the trauma experienced not only by individuals but by the family as a whole [1••, 20•, 36, 59•]. These need to be balanced with options for therapeutic support for adult survivors who themselves want support in isolation from their family.

## Conclusions

SSB is not a new or emerging form of child sexual abuse, but professional and academic discourses are changing in line with new evidence that foregrounds the complexity and multifaceted nature of this issue. Professional, practice and academic (re)discovery of SSB means that it can be explored in a nuanced way with a dual focus on both the family system and the abusive behaviour. Research, albeit limited, indicates that SSB can have significant and life course impacts on the family system in general and the victim specifically [1••, 2••], which means that perspectives, policies and interventions need to be reframed to focus on the ecosystem within which the problematic behaviour occurs rather than focusing solely on the behaviour, actions and responses of individuals within the family.
